# Rhabdomyolysis as a manifestation of clomipramine poisoning

**DOI:** 10.1590/1516-3180.2013.1316541

**Published:** 2013-12-01

**Authors:** Nathalie Oliveira de Santana, Aécio Flávio Teixeira de Góis

**Affiliations:** I MD. Resident Physician, Department of Medicine, Universidade Federal de São Paulo (Unifesp), São Paulo, Brazil.; II MD, PhD. Head, Emergency Department, Universidade Federal de São Paulo (Unifesp), São Paulo, Brazil.

**Keywords:** Clomipramine, Antidepressive agents, tricyclic, Rhabdomyolysis, Poisoning, Toxicology, Clomipramina, Antidepressivos tricíclicos, Rabdomiólise, Envenenamento, Toxicologia

## Abstract

**CONTEXT:**

Tricyclic antidepressive agents are widely used in suicide attempts and present a variety of deleterious effects. Rhabdomyolysis is a rare complication of such poisoning.

**CASE REPORT:**

A 55-year-old woman ingested 120 pills of 25 mg clomipramine in a suicide attempt two days before admission. After gastric lavage in another emergency department on the day of intake, 80 pills were removed. On admission to our department, she was disoriented, complaining of a dry mouth and tremors at the extremities. An electrocardiogram showed a sinus rhythm with narrow QRS complexes. Laboratory results showed high creatine phosphokinase (CK = 15,094 U/l on admission; normal range = 26 to 140 U/l), hypocalcemia, slightly increased serum transaminases and mild metabolic acidosis. The patient's medical history included depression with previous suicide attempts, obsessive-compulsive disorder, hypothyroidism and osteoporosis. She presented cardiac arrest with pulseless electric activity for seven minutes and afterwards, without sedation, showed continuous side-to-side eye movement. She developed refractory hypotension, with need for vasopressors. Ceftriaxone and clindamycin administration was started because of a hypothesis of bronchoaspiration. The patient remained unresponsive even without sedation, with continuous side-to-side eye movement and a decerebrate posture. She died two months later. Rhabdomyolysis is a very rare complication of poisoning due to tricyclic drugs. It had only previously been described after an overdose of cyclobenzaprine, which has a toxicity profile similar to tricyclic drugs.

**CONCLUSIONS:**

Although arrhythmia is the most important complication, rhabdomyolysis should be investigated in cases of clomipramine poisoning.

## Abstract (pt)

**CONTEXTO:**

Antidepressivos tricíclicos são amplamente utilizados em tentativas de suicídio e apresentam diversos efeitos deletérios, sendo a rabdomiólise uma complicação rara dessa intoxicação.

**RELATO DO CASO:**

Uma mulher de 55 anos ingeriu 120 comprimidos de clomipramina de 25 mg numa tentativa de suicídio dois dias antes da admissão. Após lavagem gástrica em outro serviço de urgência no dia da ingestão, 80 comprimidos foram retirados. Na admissão em nosso serviço, a paciente estava desorientada, queixando-se de boca seca e tremores de extremidades. O eletrocardiograma mostrou ritmo sinusal com complexos QRS estreitos. Exames laboratoriais evidenciaram aumento de creatinofosfoquinase (CK = 15.094 U/L na admissão; intervalo da normalidade = 26 a 140 U/L), hipocalcemia, discreto aumento das transaminases e leve acidose metabólica. Antecedentes pessoais incluíam depressão com tentativas de suicídio prévias, transtorno obsessivo compulsivo, hipotireoidismo e osteoporose. A paciente apresentou parada cardiorrespiratória com atividade elétrica sem pulso por sete minutos e, posteriormente, sem sedação, foi observado olhar em varredura. A paciente evoluiu com hipotensão refratária, necessitando de vasopressores. Ceftriaxone e clindamicina foram iniciados pela hipótese de broncoaspiração. A paciente permaneceu irresponsiva mesmo sem sedação, com olhar em varredura contínuo e postura descerebrada. A paciente evoluiu para óbito dois meses após. Rabdomiólise é uma complicação rara da intoxicação por tricíclicos, e só foi descrita em overdose de ciclobenzaprina, a qual tem um perfil de toxicidade semelhante aos tricíclicos.

**CONCLUSÕES:**

Apesar de as arritmias serem as complicações mais temidas, rabdomiólise deve ser investigada em casos de intoxicação por clomipramina.

## INTRODUCTION

Tricyclic antidepressants are among the most commonly used drugs in suicide attempts, along with benzodiazepines, alcohol and acetaminophen,^1^ surpassed only by analgesics.^2^ ^,^ ^3^ Around 300 people die each year in the United Kingdom due to tricyclic poisoning.^4^ Although the risk of suicide is the same between tricyclic drugs and other antidepressants, the death rates are higher when tricyclic drugs are used: 97% of all deaths due to antidepressant poisoning are caused by them.^5^

The case reported here forms an example of the range of clomipramine toxicity, and highlights an extremely rare complication: rhabdomyolysis.

## CASE REPORT

A 55-year-old woman was brought into the emergency room after reportedly ingesting 120 pills of 25 mg clomipramine in a suicide attempt two days before admission. Gastric lavage was performed a few hours after the ingestion in another emergency department, and 80 pills were removed. On admission to our service, she was awake, but disoriented, complaining of a dry mouth and tremors at the extremities. Her pupils were equal and reactive; she was dehydrated; and her vital signs were 86 bpm and 122/74 mmHg. An electrocardiogram showed a sinus rhythm with narrow QRS complexes.

Laboratory results showed high creatine phosphokinase (CK = 15,094 U/l on admission; normal range = 26 to 140 U/l), hypocalcemia (ionized calcium = 1.05 mmol/l; normal range = 1.15 to 1.32 mmol/l), slightly increased serum transaminases (alanine aminotransferase = 130 U/l, aspartate aminotransferase = 176 U/l; normal ranges are up to 31 and up to 32 U/l, respectively) and mild metabolic acidosis (bicarbonate content in venous blood = 19.5 mmol/l; normal range = 22 to 26 mmol/l). TSH and free T4 levels were within the normal ranges. Troponin I = 0.03 ng/ml (normal values are up to 0.04 ng/ml); urine analysis presented pH = 5.0. 

The patient's medical history included depression with previous suicide attempts, obsessive-compulsive disorder, hypothyroidism and osteoporosis. The psychoactive drugs that had been used to treat depression were clomipramine and fluoxetine. 

On the day of admission, the patient presented cardiac arrest with pulseless electric activity for seven minutes, which was treated in accordance with the Advanced Cardiac Life Support protocol, in the emergency department. A total of 100 mEq of sodium bicarbonate was administered. Spontaneous circulation returned, with sinus bradycardia and narrow QRS complexes, which were reversed with atropine. On the following day, after sedation was turned off, there was only a response to painful stimuli. The patient was admitted to the intensive care unit without sedation, showing continuous side-to-side eye movement. The tremors at the extremities and in the lips ceased after benzodiazepine administration. The first cranial computed tomography scan, produced two days after the cardiac arrest, showed signs of brain swelling, without cerebellar tonsil herniation.

The patient's urine was alkaline (pH = 7.0), and, since she had been receiving vigorous volume expansion since admission, acute renal failure did not occur. The creatine phosphokinase levels decreased and, one week after the ingestion, the CK level was 385 U/l.

A chest X-ray subsequent to the cardiac arrest revealed alveolar opacity in the left hemithorax and in the lower right hemithorax, and also diffuse bronchi and low PaO_2_/FiO_2_ ratio. Ceftriaxone and clindamycin administration was started because of a hypothesis of bronchoaspiration. The patient developed refractory hypotension, with the need for vasopressors.

Even after cessation of midazolam and fentanyl administration, the patient remained unresponsive, with continuous side-to-side eye movement and a decerebrate posture. Midazolam use was resumed, and was maintained continuously thereafter, in order to inhibit status epilepticus. The cranial computed tomography scan was repeated 48 hours after the first one, and no changes were observed. After it had been determined that the neurological prognosis was unfavorable due to anoxic brain damage, palliative care was instituted, and the patient died two months later.

## DISCUSSION

Tricyclic toxicity is due to four main pharmacological properties: anticholinergic effect, noradrenaline reuptake inhibition, alpha adrenergic receptor blockade and quinidine-like effects that block sodium channels.^6^ Despite conflicting data,^7^ there seem to be differences in the toxic levels of tricyclic drugs, since it has been described that clomipramine, amitriptyline, imipramine and trimipramine present higher death rates.^8^ ^,^ ^9^

Although data concerning the minimum toxic dose are scarce, some studies have correlated moderate toxicity with doses of 600 to 750 mg of clomipramine, while doses above 750 mg have been correlated with severe complications.^10^ ^,^ ^11^ Our patient ingested a higher dose, (3,000 mg) that was capable of causing severe poisoning, even after gastric lavage. Decontamination brings benefits for up to one hour after ingestion, and there is no difference between gastric lavage and use of activated charcoal.^12^ ^,^ ^13^

Inhibition of sodium channels delays depolarization, not only in the myocardium, but also in the conduction system. However, the incidence of severe dysrhythmias is low, ranging from 1.3% to 2.6%.^14^ ^,^ ^15^ QRS duration (> 0.16 seconds) is a better predictor of ventricular dysrhythmias and seizures than is tricyclic plasma level itself,^16^ and it is possible for a patient to present normal QRS duration in spite of a high tricyclic plasma level,^17^ such as we have described in this case.

Late toxicity has been reported, mainly involving conduction disturbance, dysrhythmias and even death after clinical improvement and stabilization.^18^ ^-^ ^20^ Nonetheless, it is not known whether these late effects are due to direct toxicity or to complications due to hospitalization.

It is important to highlight that rhabdomyolysis is a very rare complication of tricyclic antidepressive agents,^21^ and its physiopathology has not been clarified yet (Table 1). It has been described after cyclobenzaprine overdose, which has a range of toxicity similar to that of tricyclic antidepressant overdoses.^22^ ^,^ ^23^ Furthermore, there have been a few cases of serotonin syndrome that presented with rhabdomyolysis after an overdose of clomipramine and moclobemide.^24^ ^,^ ^25^ In all of these cases, the treatment consists of removing the drug, which may require dialysis, and clinical support.

**Table 1:** Description of database search strategies

Database	Search strategy	Results
Medline (Medical Literature Analysis and Retrieval System Online - via PubMed)	((antidepressive agents, tricyclic) OR Clomipramine) AND Rhabdomyolysis Filters: Case Reports	21 citations (2 related)
Lilacs (Literatura Latino-Americana e do Caribe em Ciências da Saúde - via Bireme)	((antidepressive agents, tricyclic) OR (antidepressivos tricíclicos) OR Clomipramine OR Clomipramina) AND (Rhabdomyolysis OR Rabdomiólise OR Rabdomiólisis) Filters: Case Reports	None
Embase (Excerpta Medica Database - via Elsevier)	#tricyclic antidepressant agent OR #clomipramine AND #rhabdomyolysis	13 citations (2 related)

Search date: January 30, 2013.

## CONCLUSION

Tricyclic drugs are widely used in suicide attempts and present several toxic effects. Even though dysrhythmia is the most worrisome complication, rhabdomyolysis should be remembered and investigated in cases of clomipramine poisoning.
